# Unveiling the interplay between methylglyoxal and plant growth under abiotic adversity via a red‐emissive probe operated by a ring‐closure mechanism

**DOI:** 10.1002/smo2.70106

**Published:** 2026-09-21

**Authors:** Yu‐Long Li, Xin‐Hui‐Zi Li, Ting‐Ting Liu, Yuan Qiu, Xiao‐Gang Luo, Qi Sun, Shu‐Jing Yu

**Affiliations:** ^1^ Hubei Key Laboratory of Novel Reactor and Green Chemical Technology School of Chemistry and Environmental Engineering Wuhan Institute of Technology Wuhan China; ^2^ National Pesticide Engineering Research Center (Tianjin) College of Chemistry Nankai University Tianjin China

**Keywords:** abiotic stresses, *Arabidopsis thaliana*, fluorescent probes, methylglyoxal, ring‐closure mechanisms

## Abstract

Methylglyoxal (MGO) functions as a key signaling molecule in plants, regulating plant growth, development, and stress responses, particularly under abiotic stress conditions. Despite its recognized importance, direct evidence linking MGO dynamics to plant growth under abiotic stress remains limited. To address this gap, we developed a benzoindole‐derived fluorescent probe **BH‐PDN**, which employs o‐phenylenediamine as the recognition group to enable sensitive detection of MGO through a specific ring‐closing reaction, accompanied by a pronounced red turn‐on fluorescence response (20.4‐fold enhancement). **BH‐PDN** exhibited exceptional detection capabilities, including a large Stokes shift (255 nm) and low detection limit (78 nM), allowing fluorescence visualization of exogenous and endogenous MGO‐associated changes in living cells, zebrafish, and *Arabidopsis thaliana*. Notably, **BH‐PDN** imaging revealed pronounced increases in MGO‐associated fluorescence in *Arabidopsis roots* under salt, extreme‐temperature, and drought stress, accompanied by reduced root elongation. These findings support a close association between stress‐associated MGO changes and reduced root elongation. Thus, **BH‐PDN** serves as a reliable optical window for dissecting the MGO–growth interplay, enabling real‐time interrogation of stress adaptation pathways.

## Graphical abstract

**BH‐PDN** is a red‐emissive fluorescent probe that enables methylglyoxal (MGO) detection via a ring‐closure‐triggered turn‐on response. Probe‐based imaging uncovered prominent MGO buildup in *Arabidopsis thaliana* roots exposed to salinity, extreme temperatures and drought. These results confirm a close link between stress‐induced MGO accumulation and repressed root‐growth performance.

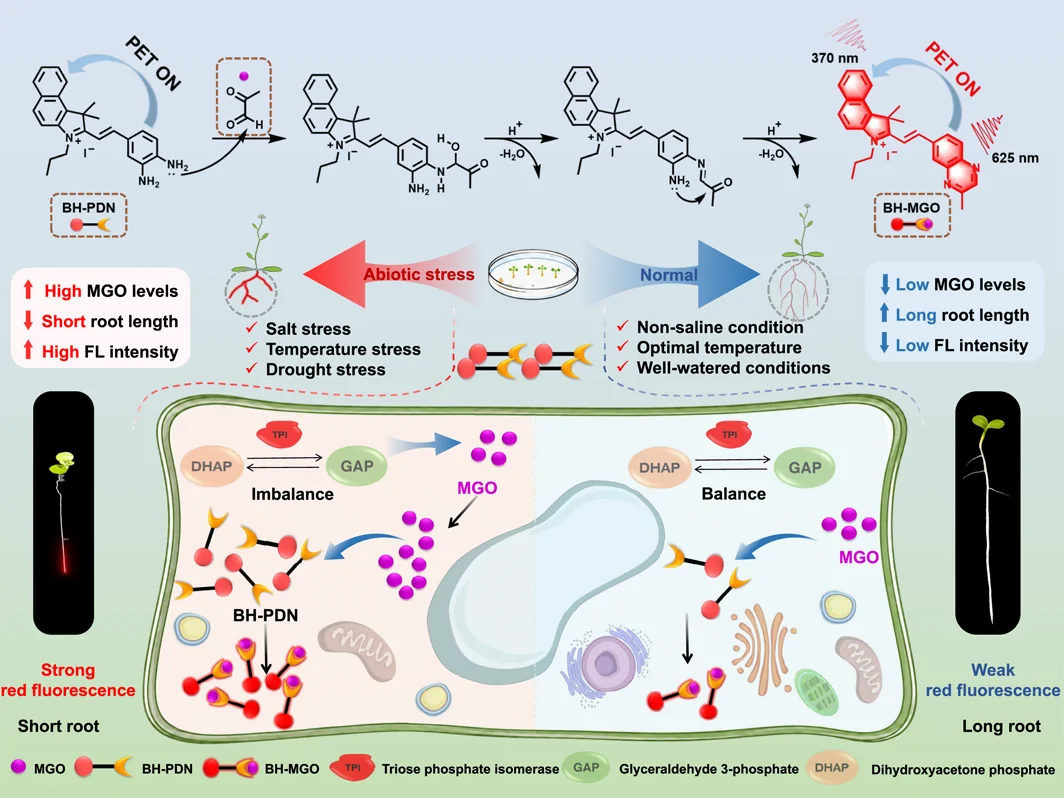

## 1 INTRODUCTION

Methylglyoxal (MGO), an active α,β‐dicarbonyl ketoaldehyde, is endogenously produced through metabolic pathways, including the catabolism of glucose, alcohols and proteins, in both animals and plants.[^1^, ^2^, ^3^, ^4^] Under physiological conditions, plants maintain MGO at steady‐state concentrations of approximately 30–75 μM via spontaneous decomposition of methylglyoxal‐phosphate, where it functions as a regulator of plant growth, development, and signaling pathways.[^5^, ^6^, ^7^, ^8^, ^9^, ^10^] Under abiotic stresses such as salinity, drought, and low temperature, MGO levels can increase by approximately 2 to 6‐fold, although the extent varies with plant species, tissue type, stress intensity, and exposure duration.[^11^, ^12^, ^13^, ^14^] including suppression of seed germination, impairment of root system morphogenesis, and disruption of photosynthetic electron transport.[^15^, ^16^, ^17^] Consequently, the development of an effective molecular probe for real‐time MGO monitoring is vital for predicting plant stress resilience and optimizing growth management strategies.

Recent studies have further highlighted fluorescence‐based molecular imaging for visualizing stress‐responsive processes in plants,[^18^, ^19^, ^20^, ^21^, ^22^] including non‐invasive real‐time monitoring of bioactive molecules with high sensitivity and spatial resolution.[^23^, ^24^, ^25^, ^26^, ^27^] Although various MGO fluorescent probes have been widely employed in cellular and animal studies, their application in plant growth studies remains unexplored (Table S1).[^28^, ^29^, ^30^, ^31^, ^32^, ^33^, ^34^, ^35^, ^36^] Our previous work reported two MGO fluorescence probes utilizing coumarin derivatives as the fluorophore and o‐phenylenediamine (OPD) as the specific recognition group for MGO detection.[^37^, ^38^] Both probes were successfully monitored MGO levels in *Arabidopsis thaliana*, revealing that drought stress induces MGO accumulation and subsequently root growth inhibition. However, these probes still exhibit limitations that need to be addressed, including poor solubility, low fluorescence enhancement, and insufficient elucidation of the relationship between MGO levels and the salt or temperature stress.

**SCHEME 1:**
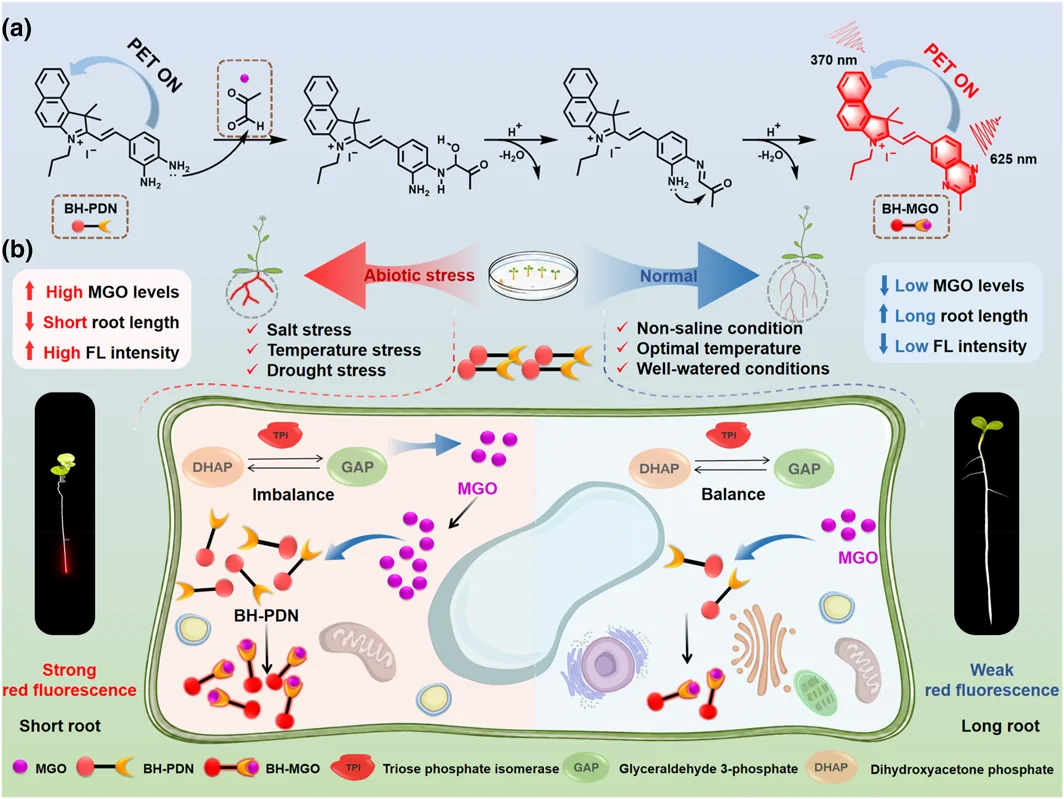
(a) Proposed reaction mechanism of probe **BH‐PDN** with MGO. (b) Visualization of MGO‐associated fluorescence changes in plant roots under abiotic stress. MGO, methylglyoxal.

To address these limitations, we rationally designed **BH‐PDN**, a novel MGO fluorescent probe featuring: (1) a cyanine derivative scaffold for enhanced aqueous solubility and high quantum yield; and (2) an OPD recognition group for specific MGO detection. As expected, the sensing mechanism of probe **BH‐PDN** involves the OPD moiety undergoing nucleophilic addition and subsequent elimination reactions with MGO, resulting in the formation of a 2‐methylquinoxaline ring accompanied by a corresponding red fluorescence turn‐on response (Scheme 1). Furthermore, **BH‐PDN** demonstrates selective and sensitive detection of MGO with prominent fluorescence enhancement in water solution containing only 0.1% N,N‐dimethylformamide (DMF), aligning well with our anticipated performance. Collectively, these properties establish **BH‐PDN** as a versatile fluorescent probe for the real‐time visualization and monitoring of endogenous and exogenous MGO in living cells, zebrafish, and *A. thaliana*. Most importantly, **BH‐PDN** was employed for in vivo imaging and relative fluorescence intensity analysis of MGO‐associated changes in *A. thaliana* roots subjected to various abiotic stress, showing that increased MGO‐associated fluorescence under abiotic stress is associated with inhibited root elongation. Overall, our findings identify MGO as a promising biochemical indicator of plant abiotic stress and establish **BH‐PDN** as a reliable molecular tool for the real‐time visualization and monitoring of stress‐induced MGO dynamics.

## 2 EXPERIMENTAL

### 2.1 Synthesis of **BH‐PDN**

All chemicals used were purchased from Shanghai Titan Technology Co. The synthesis of the probe **BH‐PDN** was followed a detailed route described in Scheme S1. All intermediates and **BH‐PDN** were characterized via ^1^H NMR; (Bruker Avance III, 400 MHz), ^13^C NMR (Bruker Avance III, 101 MHz) and high‐resolution mass spectrometry (HRMS; Agilent 1100), as shown in Figures S1–S9.

### 2.2 Standard spectral measurement

**BH‐PDN** was first dissolved in DMF to prepare a 10 mM stock solution and then diluted 1000‐fold with phosphate‐buffered saline (PBS, pH 7.4) to a final concentration of 10 μM; the final medium therefore contained 0.1% (v/v) DMF. All other analytes were used with a concentration of 100 μM. UV‐vis and fluorescence spectra of the samples reacted with MGO at different time intervals (0–60 min) were recorded at 37°C via a Spectramax M2 enzyme labeler in a 1 mL four‐pass quartz cuvette (*λ*_ex_ = 370 nm).

### 2.3 In vitro imaging

A549 cells were cultivated as reported in our previous work.[^39^, ^40^] Fluorescence imaging experiments were carried out to examine the performance of **BH‐PDN** toward both exogenous and endogenous MGO in A549 cells. For exogenous MGO detection, cells were incubated with MGO (0, 50, 100, or 200 μM) for 30 min, rinsed with PBS, and then with **BH‐PDN** (10 μM) for an additional 30 min before fluorescence observation in the red channel (600–650 nm) on an inverted fluorescence microscope (Olympus IX71). For endogenous MGO imaging, curcumin (0, 50, 100, or 200 μM) was used to induce endogenous MGO production, after which the cells were subjected to the same incubation and imaging procedure.

### 2.4 Zebrafish imaging

The applicability of **BH‐PDN** for in vivo MGO imaging was further examined in zebrafish larvae (5–10 days). Both endogenous and exogenous MGO imaging experiments were conducted following the same treatment procedures used for cells. For each treatment, 5–10 larvae were analyzed in parallel, and fluorescence images were acquired after tricaine anesthesia.

### 2.5 *A. thaliana* imaging

To evaluate the applicability of **BH‐PDN** for monitoring MGO in plants, fluorescence imaging experiments were performed using *A. thaliana*.[^41^, ^42^, ^43^, ^44^] For exogenous MGO imaging, *Arabidopsis* roots were pretreated with MGO for 30 min, followed by incubation with **BH‐PDN** (10 μM) for an additional 30 min before fluorescence imaging in the red channel. Untreated seedlings served as the control. To verify the specificity of the probe, seedlings were preincubated with N‐acetyl‐L‐cysteine (NAC, 100 μM) for 30 min prior to MGO treatment (100 μM, 30 min), followed by incubation with **BH‐PDN** (10 μM) for 30 min and fluorescence imaging.

To investigate the relationship between MGO accumulation and plant growth under abiotic stress, *Arabidopsis* seedlings were grown on half‐strength Murashige and Skoog (1/2 MS) medium supplemented with different treatments. The control group was maintained on standard 1/2 MS medium, while the remaining groups were cultured on medium containing 100 μM MGO, 200 mM D‐mannitol (drought stress), 50 or 100 mM NaCl (salt stress), or maintained at 4 or 30°C (temperature stress). Following 7 days of cultivation in a controlled growth chamber, root length was measured to evaluate plant growth. Subsequently, all seedlings were stained with **BH‐PDN** (10 μM) for fluorescence imaging to monitor endogenous MGO levels in the roots.

## 3 RESULTS AND DISCUSSION

### 3.1 Response ability of **BH‐PDN** for MGO detection

**FIGURE 1:**
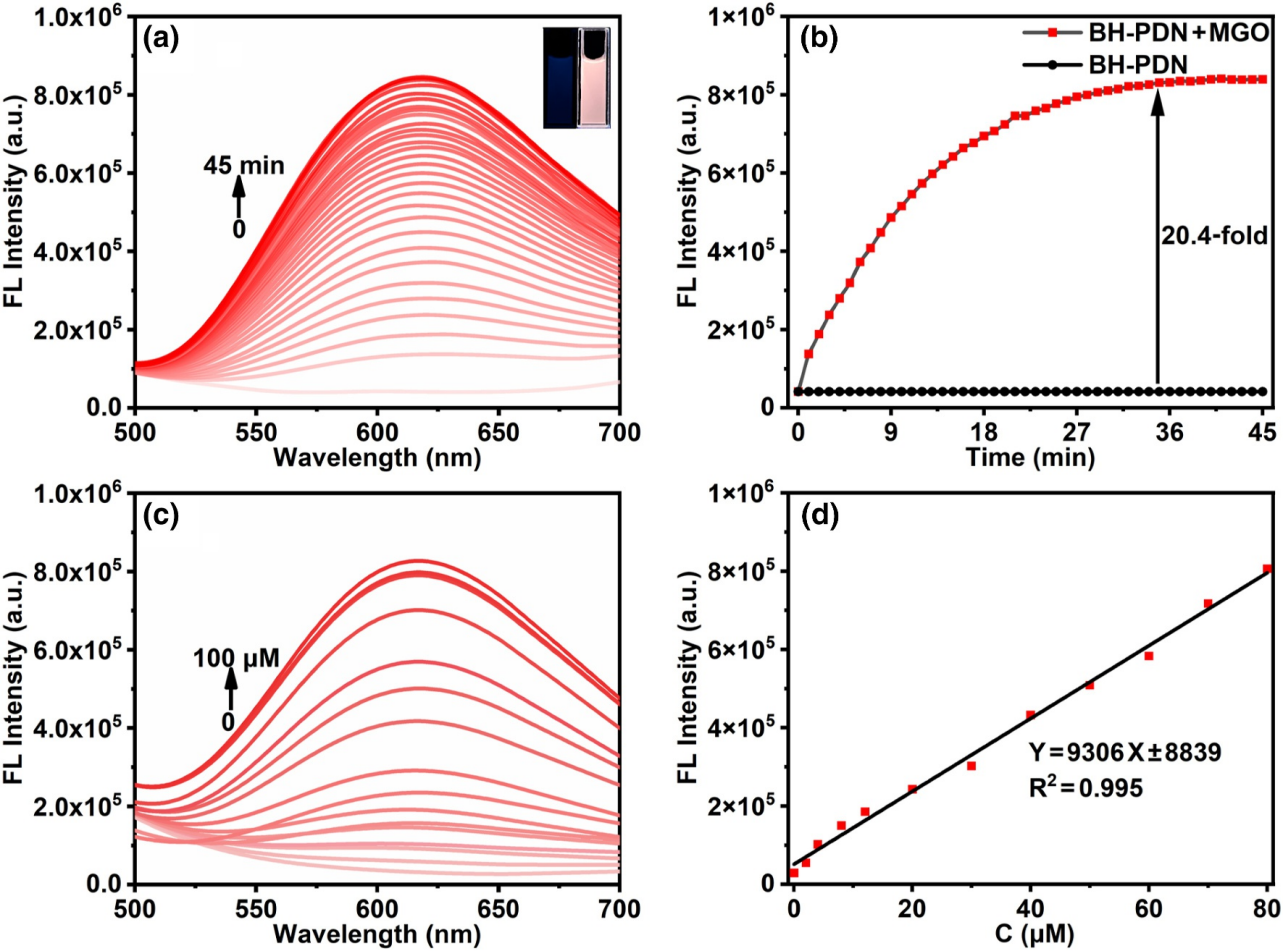
(a) Time‐dependent fluorescence response of **BH‐PDN** (10 μM) to MGO (100 μM) in 45 min. (b) Comparative fluorescence intensity at 625 nm for **BH‐PDN** (10 μM) before and after MGO (100 μM) treatment for 45 min. (c) Concentration‐dependent fluorescence intensity changes of **BH‐PDN** (10 μM) with increasing MGO concentrations (0–100 μM). (d) Linear correlation between fluorescence intensity at 625 nm of **BH‐PDN** and MGO concentrations (0–80 μM). All experiments were conducted in phosphate‐buffered saline buffer (pH 7.4) at 37°C. MGO, methylglyoxal.

To evaluate the detection ability of probe **BH‐PDN** toward MGO, UV‐visible and fluorescence spectra were recorded (Figure S10). With increasing incubation time with MGO, the absorption peak of **BH‐PDN** at 515 nm gradually decreased, while a new peak at 370 nm emerged and intensified, accompanied by an obvious color change from pink to yellow (Figure S10a). These results preliminarily confirmed that **BH‐PDN** enables ratiometric colorimetric detection of MGO within 45 min (Figure S10b). Meanwhile, the fluorescence spectra of **BH‐PDN** exhibited a consistent enhancement upon the addition of MGO within 45 min, demonstrating its ability for real‐time MGO analysis through a red fluorescence turn‐on response (Figure 1a). Further analysis of the fluorescence intensity at 625 nm revealed that free probe **BH‐PDN** showed excellent stability and a low fluorescence background. After incubation with MGO, the fluorescence intensity of **BH‐PDN** reached a plateau at 40 min, showing a 20.4‐fold fluorescence enhancement (Figure 1b), demonstrating its rapid and sensitive detection capability for MGO in aqueous solution. Next, the quantitative analysis capability of **BH‐PDN** for MGO was evaluated. As shown in Figure 1c, the fluorescence intensity of **BH‐PDN** increased significantly with the MGO concentration ranging from 0 to 100 μM. Furthermore, a good linear relationship (*R* ^2^ = 0.995) was observed between the fluorescence intensity at 625 nm and the MGO concentration (0–80 μM) (Figure 1d), proving that **BH‐PDN** can quantitatively detect MGO within the physiologically relevant concentration range. In addition, the detection limit of **BH‐PDN** for MGO was calculated to be 78 nM, validating its high sensitivity for MGO detection.

### 3.2 MGO sensing mechanism by **BH‐PDN**

The reaction mechanism between the probe **BH‐PDN** and MGO was investigated using high‐performance liquid chromatography (HPLC) and HRMS. Firstly, the potential reaction product **BH‐MGO** was synthesized as a standard. Then, the HPLC analysis revealed distinct peaks of the free probe **BH‐PDN** at 7.0 min and the product **BH‐MGO** at 9.0 min (Figure S11a,d), confirming their high purity and suitability as the control compounds. Compared with the free probe **BH‐PDN**, the reaction mixture of **BH‐PDN** incubated with MGO for 20 min showed a decreased peak at 7.0 min and a new peak at 9.0 min (Figure S11b), confirming the partial conversion of **BH‐PDN** to **BH‐MGO**. After 40 min of reaction, the peak at 7.0 min completely disappeared, and only a single peak at 9.0 min remained (Figure S11c), demonstrating the complete conversion of **BH‐PDN** to **BH‐MGO**. These observations correlated well with the time‐dependent experiments, indicating a complete transformation of **BH‐PDN** to **BH‐MGO** within 40 min. In addition, the HRMS analysis of the **BH‐PDN**/MGO reaction system revealed two characteristic peaks at *m/z* 370.2280 (corresponding to **BH‐PDN**) and 406.2279 (corresponding to BH‐MGO) (Figure S12), respectively, which is in good agreement with the HPLC results. These findings collectively support that probe **BH‐PDN** reacts with MGO via a proposed ring‐closure mechanism to generate **BH‐MGO**, as illustrated in the revised Scheme 1.

### 3.3 Specific selectivity and anti‐interference of **BH‐PDN** for MGO

**FIGURE 2:**
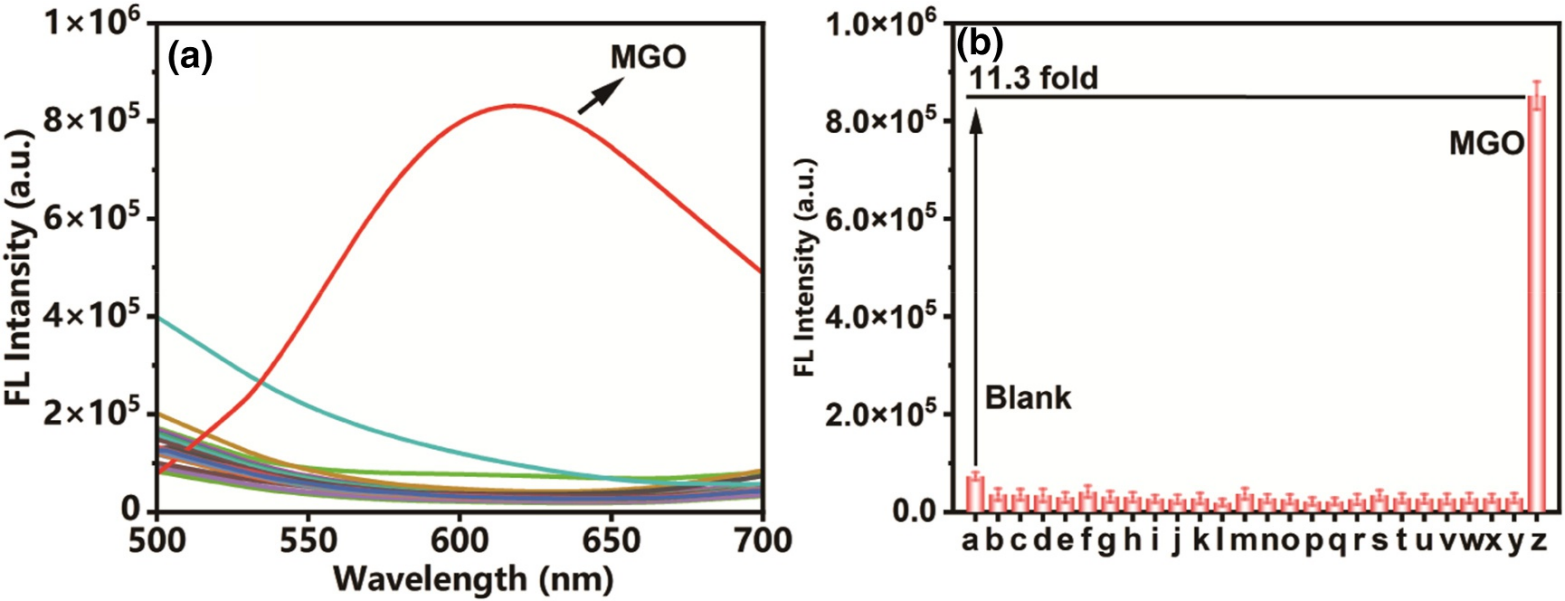
(a) Fluorescence spectra and (b) fluorescence intensities at 625 nm of **BH‐PDN** (10 μM) incubated with different analytes (100 μM each). The tested analytes were: a, blank; b, Cys; c, Hcy; d, GSH; e, Glu; f, Gly; g, S^2−^; h, HCO_3_^−^; i, NO_3_^−^; j, NO_2_^−^; k, SO_4_^2−^; l, Ba^2+^; m, Ca^2+^; n, Fe^3+^; o, Zn^2+^; p, Mg^2+^; q, oxalic acid; r, ascorbic acid; s, NO; t, HCHO; u, H_2_O_2_; v, O_2_•^−^; w, •OH; x, ^1^O_2_; y, ClO^−^; and z, methylglyoxal.

To assess the practical MGO detection capacity of **BH‐PDN** in a complex environment, the selectivity and anti‐interference experiments were conducted. As shown in Figure 2a, **BH‐PDN** exhibited a remarkable fluorescence enhancement upon exposure to MGO, while showing negligible responses to a range of potential interferents including amino acids, anions, cations, and representative reactive species (H_2_O_2_, O_2_•^−^, •OH, ^1^O_2_, and ClO^−^) Quantitative analysis at 625 nm (Figure 2b) revealed that **BH‐PDN** exhibited over 11.3‐fold higher fluorescence intensity toward MGO compared to other analytes, indicating its excellent selectivity. Furthermore, anti‐interference experiments demonstrated that **BH‐PDN** maintained stable MGO detection with fluorescence intensity variation below 10% in the presence of various interferents, including the tested reactive oxygen species at fivefold excess (Figure S13). These results reveal that **BH‐PDN** possess excellent selectivity and anti‐interference capability for MGO detection, validating its applicability in complex biological environments.

### 3.4 pH and temperature effect

To comprehensively evaluate the response capability of **BH‐PDN** toward MGO under various conditions, we examined its behavior across broad pH (1–12) and temperature (25–42°C) ranges. As shown in Figure S14a, the fluorescence intensity of **BH‐PDN** remained stable both in the presence and absence of MGO, indicating the probe's preferable stability and temperature tolerance for MGO detection. On the other hand, pH‐dependence studies revealed that free **BH‐PDN** maintained a minimal fluorescence background in physiological pH range (6–8), while showing a relatively higher fluorescence background at strong acidic (1–5) and basic (9–12) conditions (Figure S14b). Notably, in the presence of MGO, **BH‐PDN** exhibited the strongest fluorescence response at neutral pH (7–7.4), with significantly reduced signals under extreme pH conditions. These results collectively demonstrate that **BH‐PDN** is suitable for detection of MGO in biological systems, including both animal and plant systems.

### 3.5 Living cell imaging

**FIGURE 3:**
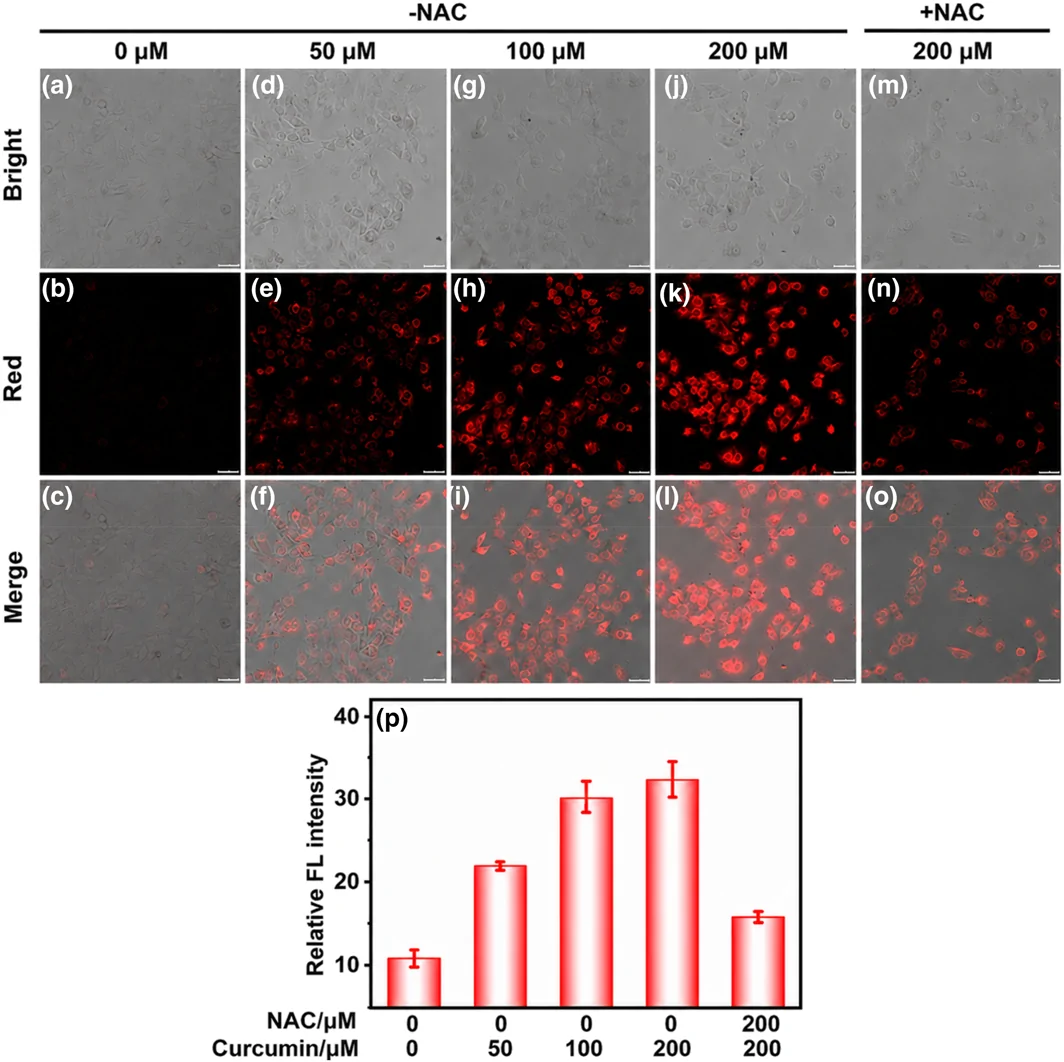
(a–l) A549 cells pretreated with curcumin (0, 50, 100, 200 μM) for 30 min, then with **BH‐PDN** (10 μM) for 30 min; (m–o) A549 cells pretreated with 200 μM curcumin for 30 min, then with 200 μM N‐acetyl‐L‐cysteine for 30 min and further with **BH‐PDN** (10 μM) for 30 min; (p) relative fluorescence intensity of the representative images. Scale bar: 200 μm.

Given the high sensitivity and selectivity of **BH‐PDN**, its capability for MGO tracking was further investigated in living cells. Initial experiments using A549 cells revealed weak red fluorescence (Figure S15b), indicating **BH‐PDN**'s ability to image and detect endogenous MGO. Dose‐dependent studies showed a corresponding increase in fluorescence intensity with rising MGO concentrations (Figure S15b,d,e), as confirmed by relative fluorescence intensity analysis (Figure S15m), thereby demonstrating that **BH‐PDN** can effectively monitor exogenous MGO levels in living cells. To specifically validate the response ability of **BH‐PDN** to MGO, a series of modulation experiments were rationally designed (Figure 3). Treatment with curcumin, a glyoxalase 1 inhibitor inducing endogenous MGO accumulation, resulted in a concentration‐dependent fluorescence enhancement (Figure 3e,h,k) compared with the controls (Figure 3b). In contrast, the treatment with NAC, a MGO quenching agent, significantly diminishes the red fluorescence signal (Figure 3n). The above relative fluorescence intensity analysis (Figure 3p) confirmed that **BH‐PDN** can specifically monitor both endogenous and exogenous MGO dynamics in living cells.

### 3.6 Zebrafish imaging

**FIGURE 4:**
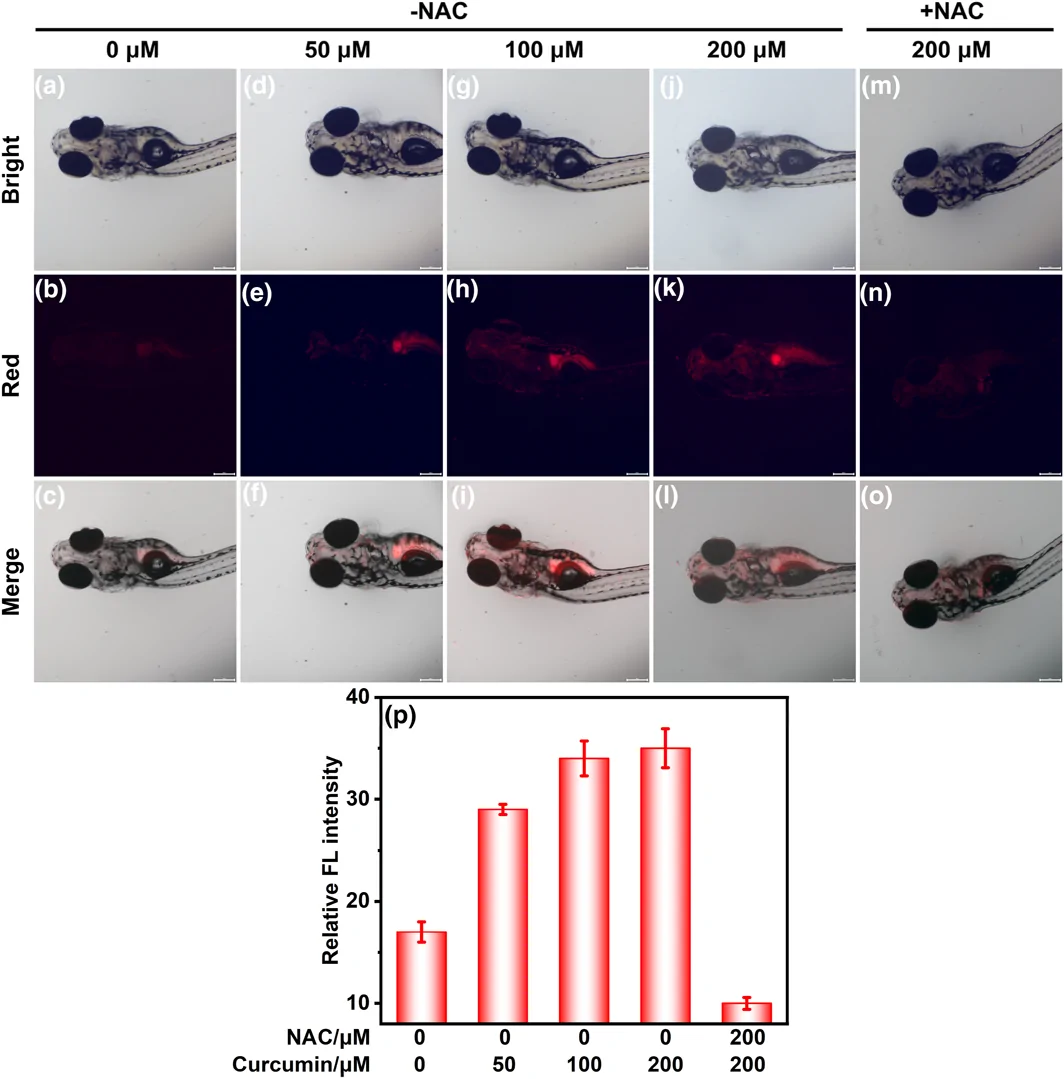
(a–l) Zebrafish pretreated with curcumin (0, 50, 100, 200 μM) for 30 min, then with **BH‐PDN** (10 μM) for 30 min; (m–o) zebrafish pretreated with 200 μM curcumin for 30 min, then with 200 μM N‐acetyl‐L‐cysteine for 30 min, and further with **BH‐PDN** (10 μM) for 30 min; (p) relative fluorescence intensity of the representative images. Scale bar: 200 μm.

Building upon the excellent MGO tracking capability of **BH‐PDN** in living cells, we extended our investigation to zebrafish models. As shown in Figure S16, fluorescence imaging revealed concentration‐dependent increase of red fluorescence in zebrafish intestines with rising MGO concentrations (Figure S16e,h,k) compared with minimal signal in the control group without MGO treatment (Figure S16b). Relative fluorescence intensity analysis (Figure S16m) further revealed the effectiveness of **BH‐PDN** to visualize exogenous MGO in vivo. Modulation of endogenous MGO levels in zebrafish showed consistent results with living cell experiments. Curcumin‐induced MGO accumulation (Figure 4e,h,k) and NAC‐mediated MGO quenching (Figure 4n) images demonstrated the specific response of **BH‐PDN** to endogenous MGO, as well as the relative fluorescence intensity data (Figure 4p). These findings collectively establish the capability of **BH‐PDN** for real‐time imaging of both endogenous and exogenous MGO in zebrafish.

### 3.7 *A. thaliana* imaging

**FIGURE 5:**
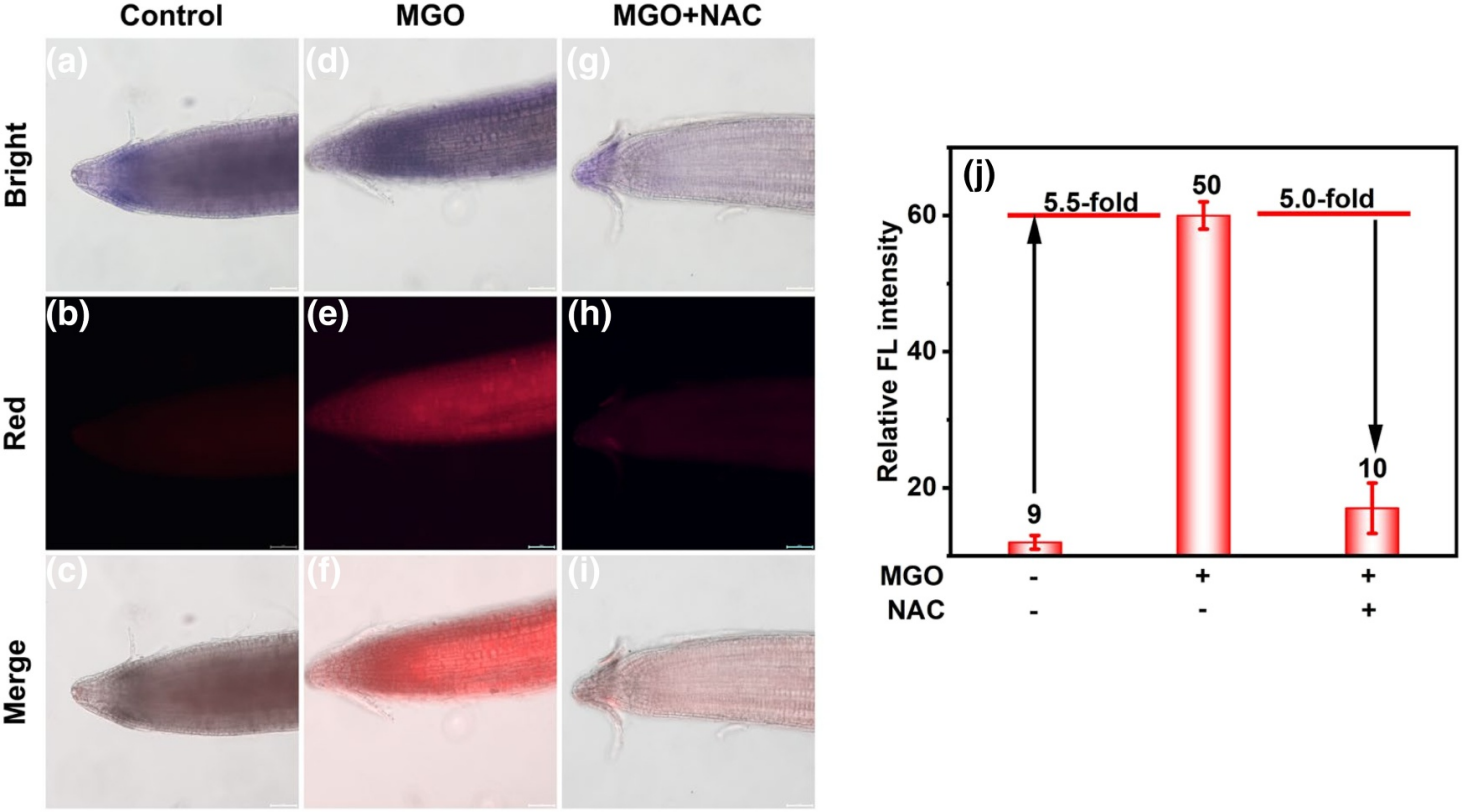
MGO detection in *Arabidopsis thaliana* root tips using **BH‐PDN**. (a–c) Roots incubated with **BH‐PDN** (10 μM) for 30 min; (d–f) roots pretreated with MGO (100 μM) for 30 min and subsequently incubated with **BH‐PDN** (10 μM) for 30 min; (g–i) roots preincubated with N‐acetyl‐L‐cysteine for 30 min, followed by MGO and **BH‐PDN** treatment; (j) relative fluorescence intensity analysis. Scale bar: 200 μm. MGO, methylglyoxal.

To further explore the potentials of **BH‐PDN** for MGO tracking in plant systems, we conducted fluorescence imaging studies in *A. thaliana* (Figure 5). *A. thaliana* root treated with **BH‐PDN** alone showed weak red fluorescence (relative intensity = 9; Figure 5b,j), indicating that **BH‐PDN** can directly detect endogenous MGO in plants. Following with MGO pretreatment, the root fluorescence signal increased significantly (relative intensity = 50; Figure 5e,j) with a 5.5‐fold enhancement compared to the endogenous MGO baseline levels (Figure 5b). Conversely, the root fluorescence maintained at near‐baseline levels after treatment with NAC (relative intensity = 10; Figure 5h,j), indicating effective suppression of MGO‐induced signal enhancement. These results support **BH‐PDN** as a useful tool for specific visualization and relative fluorescence intensity analysis of endogenous and exogenous MGO‐associated changes in plant tissues.

### 3.8 Abiotic stress studies in *A. thaliana*

**FIGURE 6:**
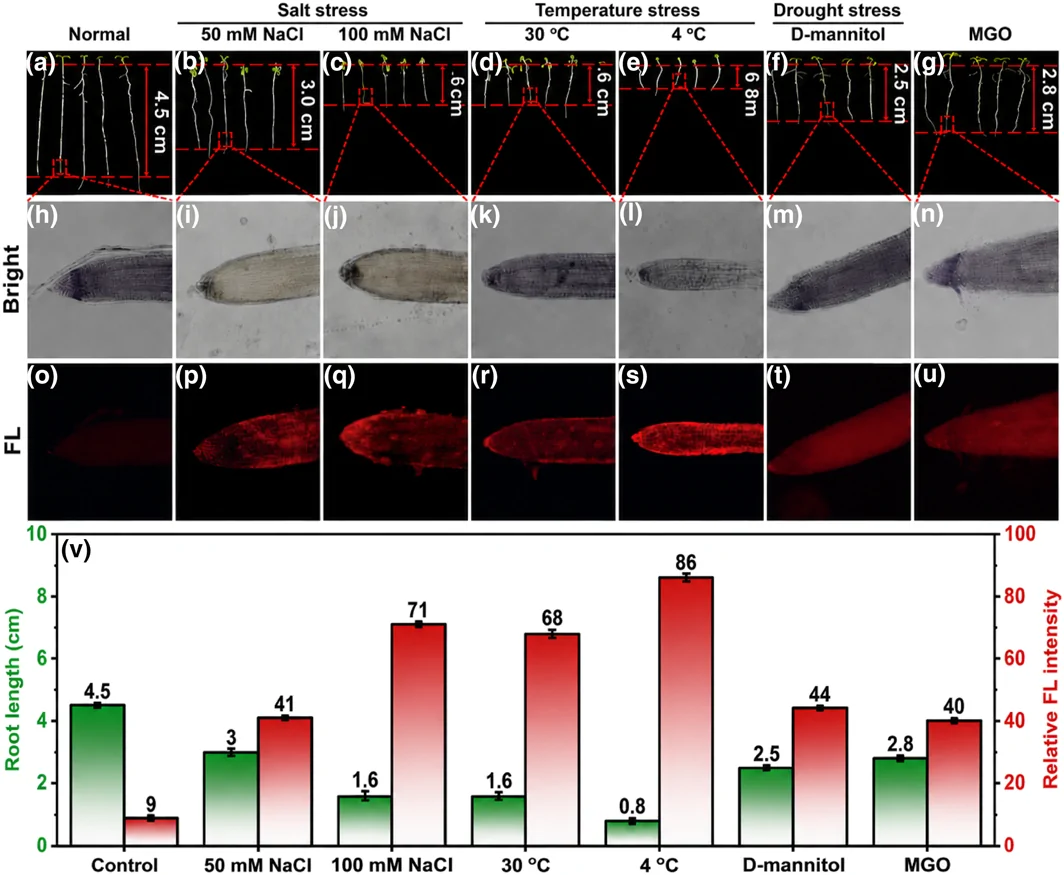
(a–g) Phenotypes of *Arabidopsis thaliana* seedlings grown for 7 days on 1/2 MS medium under different stress conditions: control, 50 mM NaCl, 100 mM NaCl, 30°C, 4°C, 200 mM D‐mannitol, and 100 μM methylglyoxal; (h–n) bright‐field images and (o–u) fluorescence images of the corresponding *A. thaliana* roots after incubation with **BH‐PDN** for 30 min; (v) root lengths and relative fluorescence intensities of the corresponding representative images. Scale bar: 200 μm.

To clarify the relationship between MGO levels and plant growth under abiotic stress, *A. thaliana* seedlings were subjected to both growth assays and MGO‐monitoring experiments (Figure 6). Under control conditions (25°C), seedlings cultured on standard medium for 7 days displayed an average root length of approximately 4.5 cm (Figure 6a,v) and showed basal MGO levels, as indicated by a weak red fluorescence signal detected using **BH‐PDN** (relative intensity = 9; Figure 6h,o,v). Under salt stress, roots grown in medium containing 50 and 100 mM NaCl were markedly shorter, with average lengths of 3.0 and 1.6 cm, respectively (Figure 6b,c,v). Correspondingly, the fluorescence intensity increased by 4.6‐ and 7.9‐fold, respectively (Figure 6i,j,p,q,v), demonstrating a strong positive correlation among salt concentration, MGO accumulation, and growth inhibition. These results indicate that salt stress simultaneously induces MGO production and suppresses root development, with both effects intensifying in a dose‐dependent manner.

Temperature stress was subsequently examined to evaluate its effects on MGO accumulation and root growth. Seedlings exposed to heat (30°C) and cold (4°C) stress exhibited reduced root lengths of 1.6 and 0.8 cm, respectively (Figure 6d,e,v), accompanied by 7.6‐ and 9.6‐fold increases in fluorescence intensity compared with the control (Figure 6k,l,r,s,v). These findings suggest that both heat and cold stress trigger MGO accumulation and inhibit root elongation, with cold stress producing a more pronounced effect.

The effect of drought stress was further investigated using D‐mannitol to induce water‐deficit conditions. Treated seedlings showed significantly reduced root growth, with an average root length of 2.5 cm, and elevated MGO levels, as indicated by a 4.8‐fold increase in fluorescence intensity compared with the control (Figure 6f,m,t,v). Consistent with the results obtained under salt and temperature stress, these findings demonstrate that drought stress also induces MGO accumulation and inhibits root growth.

To directly elucidate the role of MGO in growth suppression, seedlings were treated with exogenous MGO. As shown in Figure 6g,n,u,v, the treated roots exhibited a 4.5‐fold increase in fluorescence intensity compared with the control, confirming MGO uptake and accumulation. These seedlings also developed significantly shorter roots, with an average length of 2.8 cm, approximately 1.6‐fold shorter than that of the control, providing direct evidence that elevated MGO levels suppress root development.

### 3.9 Potential regulatory pathway of MGO on the growth of *A. thaliana*

**FIGURE 7:**
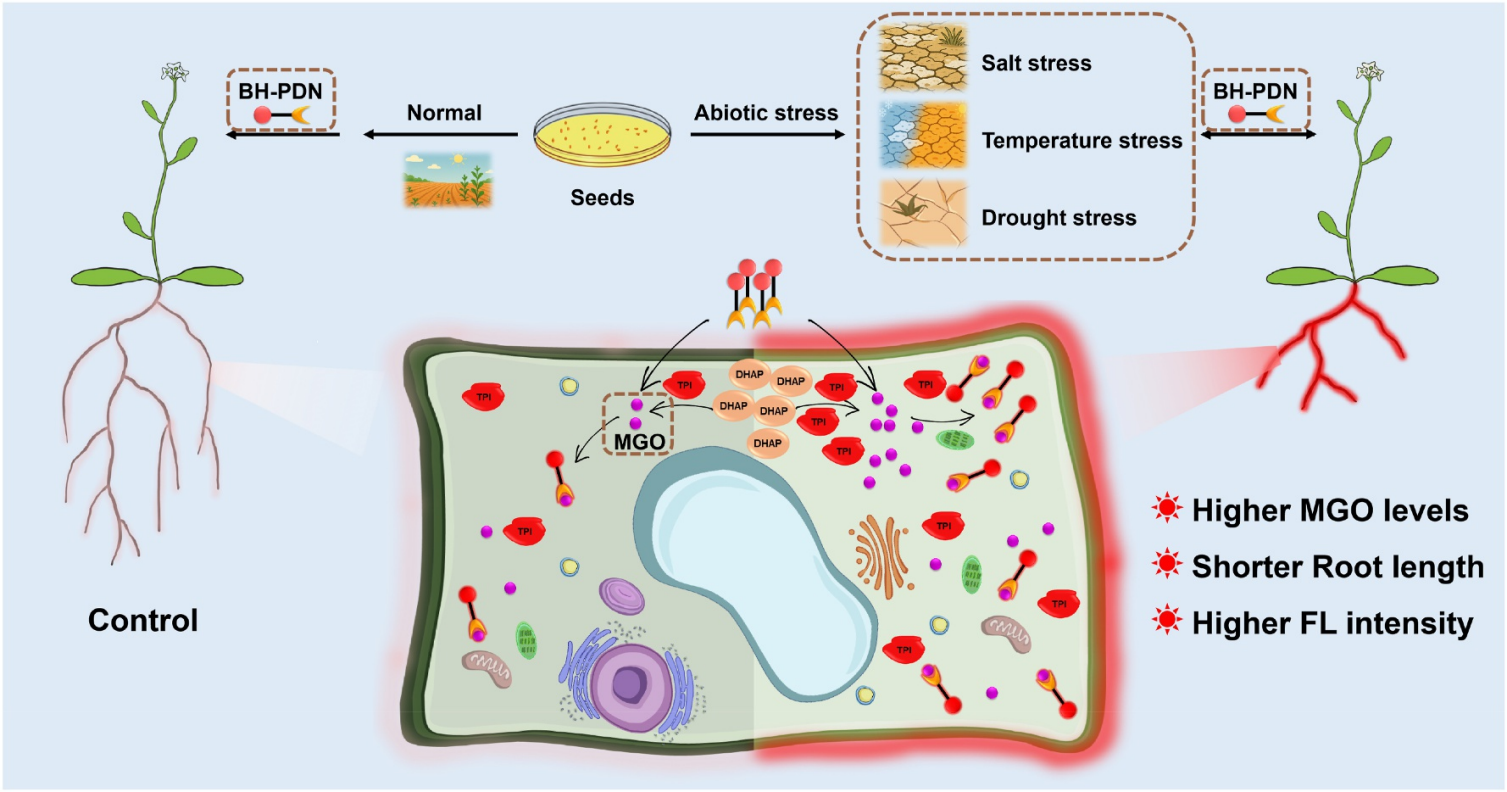
Schematic illustration of the association between stress‐associated methylglyoxal changes and reduced root elongation in plant roots under salinity, temperature, and drought stress.

As demonstrated above, abiotic stress in *A. thaliana* was accompanied by increased MGO‐associated fluorescence and reduced root elongation, and this association is summarized in Figure 7. Under normal growth conditions, plants exhibited a healthy and well‐developed root systems. However, upon exposure to abiotic stressors such as salinity, temperature, or drought, the plants showed a significant macroscopic growth alteration, most prominently with a shorter root length.

Corresponding to this phenotypic alteration, there is a series of changes in the intracellular molecular level. For instance, under stressed conditions, endogenous MGO levels were observed to be significantly higher. As depicted in Figure 7, this process involves the conversion of the glycolytic intermediate dihydroxyacetone phosphate to MGO in the presence of triosephosphate isomerase. The intracellular MGO accumulation can be visualized in vivo by the MGO‐specific fluorescent probe **BH‐PDN**. The corresponding results revealed a significantly higher fluorescence intensity in the root cells of stressed plants compared to the control, providing direct evidence for the overaccumulation of MGO.

Taken together, our findings establish a correlative pathway from abiotic stress to MGO accumulation and subsequent root growth inhibition, suggesting that MGO may act as a key negative regulatory marker mediating plant developmental responses under adverse conditions.

## 4 CONCLUSION

In this work, we developed a red‐emitting fluorescent probe **BH‐PDN** for MGO detection, based on a ring‐closing reaction between the OPD group of **BH‐PDN** and MGO which triggers a fluorescence turn‐on response. **BH‐PDN** exhibits excellent sensitivity and selectivity toward MGO, characterized by a high fluorescence enhancement (20.4‐fold), a large Stokes shift (255 nm), and a low detection limit (78 nM). Collectively, our results establish **BH‐PDN** as a valuable molecular tool for monitoring plant stress responses and investigating the underlying mechanisms of MGO accumulation under abiotic stress conditions. The **BH‐PDN** probe successfully enabled fluorescence visualization and relative fluorescence intensity analysis of exogenous and endogenous MGO‐associated changes in living cells, zebrafish and *A. thaliana*. Furthermore, **BH‐PDN** revealed significant MGO accumulation in *A. thaliana* root under various abiotic stresses (salinity, extreme temperature and drought stress), further supporting a close association that abiotic stress‐induced MGO accumulation correlates closely with root growth inhibition. These results suggest that MGO serves as a potential biomarker for abiotic stress in plants. Our findings establish **BH‐PDN** as a valuable molecular tool for monitoring plant stress response and investigating the underlying mechanisms of MGO accumulation under abiotic stress conditions.

## CONFLICT OF INTEREST STATEMENT

The authors declare no conflicts of interest.

## ETHICS STATEMENT

All zebrafish experiments were performed in accordance with the institutional guidelines for the care and use of laboratory animals formulated by the Ministry of Science and Technology of China. All experimental protocols were approved by the Institutional Animal Care and Use Committee of Wuhan Institute of Technology.

## Supporting information

Supporting Information S1

## Data Availability

The data that support the findings of this study are available from the corresponding authors upon reasonable request.
