# Supplementary material for: Unveiling the interplay between methylglyoxal and plant growth under abiotic adversity via a red‐emissive probe operated by a ring‐closure mechanism
Source: Smart Mol. 2026 Sep 21:e70106. Online ahead of print. doi: 10.1002/smo2.70106 (PMC13594573; doi:10.1002/smo2.70106)
Supplement: Supplementary file 1 [file SMO2-9999-0-s001.docx]

**Supporting Information**

**Unveiling the Interplay between Methylglyoxal and Plant Growth under Abiotic Adversity via a Red-Emissive Probe Operated by a Ring-Closure Mechanism**

*Yu-Long Li, Xin-Hui-Zi Li, Ting-Ting Liu, Yuan Qiu, Xiao-Gang Luo, Qi Sun*, and Shu-Jing Yu**

Y.-L. Li, X.-H.-Z. Li, T.-T. Liu, Y. Qiu, X.-G. Luo, Q. Sun

School of Chemistry and Environmental Engineering, Hubei Key Laboratory of Novel Reactor and Green Chemical Technology, Wuhan Institute of Technology, Wuhan 430205, China

E-mail: qisun@wit.edu.cn

S.-J. Yu

National Pesticide Engineering Research Center (Tianjin), College of Chemistry, Nankai University, Tianjin 300071, China

E-mail: yushujing@nankai.edu.cn

Table of Contents

[1. Properties of the reported MGO fluorescent probes	3](#_Toc235542606)

[2. Synthesis route of **BH-PDN** and BH-MGO	5](#_Toc235542607)

[3. NMR and HRMS	8](#_Toc235542608)

[4. UV-visible absorption spectra	12](#_Toc235542609)

[5. HPLC analysis of **BH-PDN** towards MGO	13](#_Toc235542610)

[6. HRMS analysis of **BH-PDN** reaction with MGO	13](#_Toc235542611)

[7. Interference immunity of **BH-PDN** for MGO	14](#_Toc235542612)

[8. pH and temperature effect of **BH-PDN** for MGO	14](#_Toc235542613)

[9. Living cell imaging	15](#_Toc235542614)

[10. Zebrafish imaging	16](#_Toc235542615)

[References	17](#_Toc235542616)

## 1. Properties of the reported MGO fluorescent probes

**Table S1**. Comparison of **BH-PDN** with the reported MGO probes.

| **Probe** | **λ_ex_/λ_em_**  (nm) | **Time**  (min) | **LOD**  (μM) | **Application** | **Ref.** |
| --- | --- | --- | --- | --- | --- |
|   **NI-OPD** | 380/460 | 120 | 0.06 | Cell imaging | [1] |
|   **CMFP** | 350/440 | 90 | 0.24 | Cell imaging | [2] |
|   **MEBTD** | 500/650 | 180 | 0.018 | Cell imaging | [3] |
|   **DBTPP** | 497/650 | 90 | 0.26 | Cell imaging | [4] |
|   **ISP-MGO** | 415/485 | 60 | 2.18 | Cell imaging | [5] |
|   **MGO-Naph-A** | 377/532 | 60 | 1.36 | Cell and  Zebrafish imaging | [6] |
|   **Hcy-OPD** | 570/658 | 60 min | 0.22 μM | Cell imaging | [7] |
|   **SWJT-2** | 440/674 | 15 | 0.32 | Cell imaging | [8] |
|   **Cy-DNH_2_** | 470/545 | 30 | 0.095 | Cell imaging | [9] |
|   **CNDS** | 490/590 | 90 | 0.14 | Cell, Zebrafish and  *Arabidopsis thaliana* imaging | [10] |
|   **CPDN** | 520/610 | 60 | 0.19 | Cell, Zebrafish and  *Arabidopsis thaliana* imaging | [11] |
|   **BH-PDN** | 370/625 | 40 | 0.078 | Cell, Zebrafish and  *Arabidopsis thaliana* imaging | This Work |

## 2. Synthesis route of BH-PDN and BH-MGO

**Scheme S1.** Synthetic routes of **BH-PDO**, **BH-PDN**, and **BH-MGO**.

Synthesis of Compound **C-2**

**C-1** (1054 mg, 5 mmol) was dissolved in 15 mL of anhydrous acetonitrile, and 1-iodopropane (3.5 mL, 35 mmol) was added slowly and refluxed at 80 °C for 17 h. After completion of the reaction, the reaction was cooled to room temperature, and the solvent was removed by distillation under reduced pressure to obtain a dark green solid. The solid was washed with 1 mL of methanol and 5 mL of anhydrous ether to remove impurities. The solid was dissolved in 2 mL of dichloromethane, 10 mL of petroleum ether was added, and the solvent was removed by distillation under reduced pressure to give 1590 mg of light green solid in 83% yield. ^1^H NMR (400 MHz, DMSO-*d*_6_) δ 8.40 (d, *J* = 8.3 Hz, 1H), 8.32 (d, *J* = 8.9 Hz, 1H), 8.23 (t, *J* = 9.4 Hz, 2H), 7.83-7.72 (m, 2H), 4.61 (t, *J* = 7.4 Hz, 2H), 3.00 (s, 3H), 1.96 (q, *J* = 7.4 Hz, 2H), 1.79 (s, 6H), 1.05 (t, *J* = 7.3 Hz, 3H).

Synthesis of Compound **D-2**

The temperature was controlled at 0-5 ^O^C. Concentrated nitric acid (0.375 mL) was slowly added dropwise to concentrated sulfuric acid (2.25 mL), and mixed homogeneously. Then the above mixed solution was added slowly dropwise to **D-1** (1 g, 6.13 mmol) and the solid gradually dissolved. Upon completion of the reaction, the reaction was transferred to an ice-water mixture, and the solid was precipitated to give a yellow crude product, which was then purified by column chromatography (eluent: from petroleum ether/ethyl acetate = 1/5 to 1/12, v/v) to give 1.1 g of the yellow solid, with a yield of 81%. ^1^H NMR (400 MHz, DMSO-*d*_6_) δ 10.61 (s, 1H), 9.98 (s, 1H),8.44 (s, 1H), 8.15 (d, *J* = 8.0 Hz, 1H), 7.87 (d. *J* = 8.0 Hz, 1H), 2.14 (s, 3H).

Synthesis of Compound **C-3**

Compounds **C-2** (380 mg, 1 mmol) and **D-2** (312 mg, 1.5 mmol) were dissolved in 10 mL of anhydrous acetonitrile, to which 80 μL of piperidine was added, and the solution was stirred at room temperature for 1 min, the solution changed to yellow, and then stirred at 80 ℃ for a further 1 h. At the end of the reaction, the solvent was removed by distillation under reduced pressure, and the orange-yellow solid powder was obtained. The crude product was purified by silica gel column chromatography (eluent: from dichloromethane/methanol = 200/1 to 20/1, v/v), and 0.454 g of yellow solid was obtained in 81% yield. ^1^H NMR (400 MHz, DMSO-*d*_6_) δ 10.64 (s, 1H), 8.87 (s, 1H), 8.68-8.58 (m, 2H), 8.49 (s, 1H), 8.32 (d, *J* = 9.0 Hz, 1H), 8.27-8.16 (m, 2H), 7.90 (d, *J* = 8.7 Hz, 1H), 7.86-7.73 (m, 3H), 4.86 (t, *J* = 7.4 Hz, 2H), 2.16 (s, 3H), 2.05 (s, 6H), 1.97 (q, *J* = 7.2 Hz, 2H), 1.04 (t, *J* = 7.3 Hz, 3H).

Synthesis of Compound **BH-PDO**

Compound **C-3** (380 mg, 0.67 mmol) was weighed and dissolved in 10 mL of ethyl alcohol, stirred until the solid was completely dissolved, and then 1 mL of concentrated hydrochloric acid was added and heated to 80 ℃ and refluxed for 4 h. After the reaction was completed, the reaction solution was cooled to room temperature, and the ethyl acetate was removed by distillation under reduced pressure. Then 1 mL of saturated saline was added, and the pH was adjusted to 8-10 with 15% sodium hydroxide solution, and the red crude product was extracted with ethyl acetate, and then distilled under reduced pressure to obtain the red product. The crude product was purified by column chromatography (eluent: dichloromethane/methanol = 100/1 to 10/1, v/v) to give 269 mg of red solid in 77% yield. ^1^H NMR (400 MHz, DMSO-*d*_6_) δ 8.97 (d, *J* = 2.0 Hz, 1H), 8.62 (d, *J* = 16.0 Hz, 1H), 8.55-8.31 (m, 4H), 8.28 (d, *J* = 8.9 Hz, 1H), 8.21 (d, *J* = 8.2 Hz, 1H), 8.11 (d, *J* = 9.0 Hz, 1H), 7.81 (ddd, *J* = 8.3, 6.8 Hz, 1H), 7.72 (t, *J* = 7.5 Hz, 1H), 7.51 (d, *J* = 16.0 Hz, 1H), 7.23 (d, *J* = 9.2 Hz, 1H), 4.76 (t, *J* = 7.2 Hz, 2H), 2.04 (s, 6H), 1.92 (dt, *J* = 13.3 Hz, 2H), 1.04 (t, *J* = 7.3Hz, 3H).

Synthesis of Compound **BH-PDN**

The compound **BH-PDO** (250 mg, 0.47 mmol) was dissolved in 10 mL of ethyl acetate and stirred until the solid was completely dissolved, then stannous chloride dihydrate (2134 mg, 9.48 mmol) and concentrated hydrochloric acid (1.8 mL) were added and the reaction was refluxed at 80 ^O^C for 3.5 h. At the end of the reaction, the reaction solution was cooled down to room temperature, and then the purple crude product was extracted with ethyl acetate by decompression distillation. At the end of the reaction, the reaction solution was cooled to room temperature, then ethyl acetate was removed under reduced pressure, 1 mL of saturated saline was added to it, and the pH was adjusted to 8-10 with 15% sodium hydroxide solution, then it was extracted with ethyl acetate, and then distilled under reduced pressure to obtain the purple crude product, which was then purified by column chromatography on silica gel (eluent: dichloromethane/methanol = 175/1 to 8/1, v/v), and the purple solid of 149 mg was obtained with a yield of 62%. ^1^H NMR (400 MHz, DMSO-*d*_6_) δ 8.33 (d, *J* = 8.5 Hz, 1H), 8.27-8.10 (m, 3H), 7.96 (d, *J* = 9.0 Hz, 1H), 7.81-7.69 (m, 2H), 7.65-7.59 (m, 1H), 7.48 (d, *J* = 8.2 Hz, 1H), 7.33 (s, 1H), 7.07 (d, *J* = 15.5 Hz, 2H), 6.86 (s, 2H), 6.70 (d, J = 8.3 Hz, 1H), 4.55 (m, 2H), 1.99 (s, 3H), 1.91 (s, 3H), 1.87 (m, 2H), 1.03 (t, J = 7.4 Hz, 3H); ^13^C NMR (101 MHz, DMSO-*d_6_*) δ 179.66, 172.43, 153.83, 146.68, 139.44, 136.11, 132.69, 131.08, 129.94, 129.40, 127.56, 126.93, 124.41, 123.04, 114.10, 112.97, 109.76, 103.26, 74.44, 52.63, 32.00, 29.89, 26.98, 21.55, 11.36; HRMS（ESI）calcd for C_25_H_28_N_3_ [M-I]^+^ 370.2270, found 370.2286.

Synthesis of Compound **BH-MGO**

**BH-PDN** (0.080 g, 0.16 mmol) and methylglyoxal (0.120 g, 1.2 mmol) were placed in a reaction flask, 3 mL tetrahydrofuran was added to dissolve them, heated to 60 ℃, and the reaction was reflux for 60 min. After the reaction, the solvent was removed by distillation under reduced pressure to obtain a yellow crude product. Then the crude product was purified by silica gel chromatography (eluent: petroleum ether/dichloromethane = 25/1 to 3/1, v/v) to obtain 0.063 g of yellow solid (yield 75%).

## 3. NMR and HRMS

**Figure S1. ^1^H NMR** spectrum of compound **C-2**.

**Figure S2. ^1^H NMR** spectrum of compound **D-2**.

**Figure S3. ^1^H NMR** spectrum of compound **C-3**.

**Figure S4. ^1^H NMR** spectrum of **BH-PDO**.

**Figure S5. ^1^H NMR** spectrum of **BH-PDN**.

**Figure S6. ^13^C NMR** spectrum of **BH-PDN.**

**Figure S7. HRMS** spectrum of probe **BH-PDN.**

**Figure S8. ^1^H NMR** spectrum of **BH-MGO**.

**Figure S9. HRMS** spectrum of **BH-MGO.**

## 4. UV-visible absorption spectra

**Figure S10.** (a) Time-dependent UV-vis spectral changes and (b) corresponding absorbance at 515 nm and 370 nm of **BH-PDN** (10 μM) upon reaction with MGO (100 μM) in PBS buffer (pH 7.4, 37 °C).

## 5. HPLC analysis of BH-PDN towards MGO

**Figure S11.** HPLC analysis of (a) **BH-PDN**, (b) the reaction mixture of **BH-PDN** (10 μM) with MGO (100 μM) after 20 min, (c) the reaction mixture after 40 min, and (d) **BH-MGO** in PBS buffer (pH 7.4, 37 °C)

## 6. HRMS analysis of BH-PDN reaction with MGO

**Figure S12.** HRMS analysis of the reaction product between **BH-PDN** and MGO.

## 7. Interference immunity of BH-PDN for MGO

**Figure S13.** (a) Fluorescence spectra and (b) fluorescence intensities at 625 nm of **BH-PDN** (10 μM) toward MGO (100 μM) in the presence of various potential interferents (500 μM each). The tested interferents were: a, blank (MGO alone); b, Cys; c, Hcy; d, GSH; e, Glu; f, Gly; g, S²⁻; h, HCO₃⁻; i, NO₃⁻; j, NO₂⁻; k, SO₄²⁻; l, Ba²⁺; m, Ca²⁺; n, Fe³⁺; o, Zn²⁺; p, Mg²⁺; q, K⁺; r, NO; s, HCHO; t, ascorbic acid; u, oxalic acid; v, H₂O₂; w, O₂•⁻; x, •OH; y, ¹O₂; and z, ClO⁻.

## 8. pH and temperature effect of BH-PDN for MGO

**Figure S14.** Fluorescence intensity at 625 nm of **BH-PDN** (10 μM) with and without MGO (100 μM) under different (a) temperature (25–42 °C) and (b) pH (1–12).

## 9. Living cell imaging

**Figure S15.** (a-l) A549 cells pretreated with MGO (0, 50, 100, 200 μM) for 30 min and then with **BH-PDN** (10 μM) for 30 min (a–l); relative fluorescence intensity of representative images (m). Scale bar: 200 μm.

## 10. Zebrafish imaging

**Figure S16.** (a–l) Zebrafish pretreated with MGO (0, 50, 100, 200 μM) for 30 min and then with **BH-PDN** (10 μM) for 30 min; (m) relative fluorescence intensity of representative images. Scale bar: 200 μm.

## References

[1] M. Yang, J. Fan, J. Zhang, J. Du, X. Peng, *Chem. Sci.* **2018**, *9*, 6758–6764.
[2] H. Wang, Y. Xu, L. Rao, C. Yang, H. Yuan, T. Gao, X. Chen, H. Sun, M. Xian, C. Liu, *Anal. Chem.* **2019**, *91*, 5646–5653.
[3] C. Ding, F. Wang, Y. Dang, Z. Xu, L. Li, Y. Lai, H. Yu, Y. Luo, R. Huang, A. Zhang, *Anal. Chem.* **2019**, *91*, 15577–15584.
[4] Y. Dang, F. Wang, L. Li, Y. Lai, Z. Xu, Z. Xiong, A. Zhang, Y. Tian, C. Ding, W. Zhang, *Chem. Commun.* **2020**, *56*, 707–710.
[5] Z. Liang, Z. Jiang, C. Zhang, Z. Liu, *J. Mol. Struct.* **2023**, *1285*, 135484.
[6] Z. Luo, Z. Zhu, T. Zhang, H. Jiang, N. Huang, F. Liang, Z. Wang, Y. Li, X. He, S. Qian, *Analyst* **2022**, *147*, 4949–4953.
[7] Z. Wang, Y. Bian, C. Liu, S. He, L. Zhao, X. Zeng, *Chem. Commun.* **2022**, *58*, 6453–6456.
[8] W. Xu, S. Liu, W. Cao, H. Xiong, *Sens. Diagn.* **2024**, *3*, 147–152.
[9] H. Xu, X. R. Liu, Z. H. Cai, J. Zheng, Y. W. Wang, Y. Peng, *Org. Biomol. Chem.* **2022**, *20*, 4782–4786.
[10] T. Liu, Z. Cheng, Y. Wu, Y. Qiu, X. Luo, G. Liu, Q. Sun, *Adv. Agrochem.* **2025**, *4*, 90–96.
[11] Z. Cheng, T. Liu, Y. Wu, Y. Qiu, G. Liu, Q. Sun, *Sens. Actuators B Chem.* **2025**, *423*, 136806.
